# Supplementary material for: Exploration of Metabolic Biomarkers Linking Red Meat Consumption to Ischemic Heart Disease Mortality in the UK Biobank
Source: Nutrients. 2023 Apr 13;15(8):1865. doi: 10.3390/nu15081865 (PMC10142709; doi:10.3390/nu15081865)

Supplementary Tables and Figures

Supplementary Table S1. Association of meat consumption and IHD 2

Supplementary Table S2. Distribution of 167 metabolic biomarkers 3

Supplementary Table S3: Overall association of 167 metabolic biomarkers with ischemic heart disease 7

Supplementary Table S4: Association of 10 metabolic biomarkers with red meat consumption and with ischemic heart disease in women 11

Supplementary Figure S1: Flow chart of eligible study participants in analyses 12

Supplementary Figure S2: Association of all metabolic biomarkers with unprocessed red meat and processed meat consumption (please see the separate pdf file)

Supplementary Table S1. Association of meat consumption and IHD

	OR, 95% CI_overall	p_overall	OR, 95% CI_men	p_men	OR, 95% CI_women	p_women
Unprocessed red meat consumption	1.04 (1.02, 1.07)	0.0007	1.05 (1.02, 1.07)	0.00042	1.01 (0.95, 1.08)	0.69
Processed meat consumption	1.05 (1.02, 1.07)	0.0005	1.05 (1.02, 1.07)	0.00042	1.01 (0.94, 1.09)	0.73

Footnote: Multivariable logistic regression adjusted for age, sex, ethnicity, TDI, education, physical activity, smoking status and alcohol status, medications use, and baseline disease status was used

Supplementary Table S2. Distribution of 167 metabolic biomarkers

Metabolites	Mean	SD
X3 Hydroxybutyrate	5.78E-02	5.89E-02
Acetate	1.68E-02	3.25E-02
Acetoacetate	1.26E-02	1.21E-02
Acetone	1.42E-02	5.55E-03
Glutamine	5.30E-01	7.96E-02
Histidine	6.42E-02	1.04E-02
Glycine	1.62E-01	6.38E-02
Alanine	2.96E-01	7.64E-02
Leucine	1.01E-01	2.77E-02
Isoleucine	4.98E-02	1.75E-02
Valine	2.03E-01	4.19E-02
Phenylalanine	4.50E-02	1.08E-02
Tyrosine	6.14E-02	1.39E-02
Total Concentration of Branched Chain Amino Acids (Leucine+Isoleucine+Valine)	3.53E-01	8.38E-02
Apolipoprotein A1	1.44E+00	2.41E-01
Apolipoprotein B	8.40E-01	1.99E-01
Average Diameter for HDL Particles	9.64E+00	2.35E-01
Average Diameter for LDL Particles	2.39E+01	8.80E-02
Average Diameter for VLDL Particles	3.86E+01	1.34E+00
Total Concentration of Lipoprotein Particles	1.66E-02	2.52E-03
Total Lipids in Chylomicrons and Extremely Large VLDL	2.31E-01	2.06E-01
Total Lipids in HDL	2.98E+00	6.37E-01
Total Lipids in IDL	1.22E+00	2.88E-01
Total Lipids in LDL	2.45E+00	5.93E-01
Total Lipids in Large HDL	6.45E-01	3.22E-01
Total Lipids in Large LDL	1.56E+00	3.74E-01
Total Lipids in Large VLDL	3.28E-01	1.75E-01
Total Lipids in Lipoprotein Particles	8.75E+00	1.64E+00
Total Lipids in Medium HDL	1.02E+00	2.23E-01
Total Lipids in Medium LDL	6.10E-01	1.63E-01
Total Lipids in Medium VLDL	5.67E-01	2.04E-01
Total Lipids in Small HDL	1.15E+00	1.56E-01
Total Lipids in Small LDL	2.81E-01	6.45E-02
Total Lipids in Small VLDL	4.08E-01	1.29E-01
Total Lipids in VLDL	2.09E+00	8.56E-01
Total Lipids in Very Large HDL	1.68E-01	7.95E-02
Total Lipids in Very Large VLDL	2.03E-01	1.34E-01
Total Lipids in Very Small VLDL	3.58E-01	8.83E-02
Free Cholesterol in Chylomicrons and Extremely Large VLDL	2.54E-02	1.95E-02
Free Cholesterol in HDL	2.90E-01	7.29E-02

Free Cholesterol in IDL	2.19E-01	5.62E-02
Free Cholesterol in LDL	4.58E-01	1.20E-01
Free Cholesterol in Large HDL	6.68E-02	3.75E-02
Free Cholesterol in Large LDL	2.92E-01	7.69E-02
Free Cholesterol in Large VLDL	4.62E-02	2.47E-02
Free Cholesterol in Medium HDL	8.58E-02	2.45E-02
Free Cholesterol in Medium LDL	1.17E-01	3.19E-02
Free Cholesterol in Medium VLDL	7.81E-02	2.93E-02
Free Cholesterol in Small HDL	1.13E-01	1.60E-02
Free Cholesterol in Small LDL	4.88E-02	1.24E-02
Free Cholesterol in Small VLDL	5.80E-02	1.87E-02
Free Cholesterol in VLDL	2.89E-01	1.07E-01
Free Cholesterol in Very Large HDL	2.36E-02	7.26E-03
Free Cholesterol in Very Large VLDL	2.38E-02	1.43E-02
Free Cholesterol in Very Small VLDL	5.78E-02	1.48E-02
Concentration of Chylomicrons and Extremely Large VLDL Particles	1.71E-06	1.53E-06
Concentration of HDL Particles	1.50E-02	2.41E-03
Concentration of IDL Particles	3.08E-04	7.63E-05
Concentration of LDL Particles	1.18E-03	2.80E-04
Concentration of Large HDL Particles	1.39E-03	7.64E-04
Concentration of Large LDL Particles	7.26E-04	1.75E-04
Concentration of Large VLDL Particles	1.02E-05	5.50E-06
Concentration of Medium HDL Particles	3.81E-03	9.20E-04
Concentration of Medium LDL Particles	2.91E-04	7.53E-05
Concentration of Medium VLDL Particles	3.43E-05	1.22E-05
Concentration of Small HDL Particles	9.56E-03	1.29E-03
Concentration of Small LDL Particles	1.68E-04	3.72E-05
Concentration of Small VLDL Particles	3.89E-05	1.27E-05
Concentration of VLDL Particles	1.44E-04	4.41E-05
Concentration of Very Large HDL Particles	2.32E-04	9.48E-05
Concentration of Very Large VLDL Particles	3.48E-06	2.27E-06
Concentration of Very Small VLDL Particles	5.49E-05	1.37E-05
Cholesteryl Esters in Chylomicrons and Extremely Large VLDL	3.13E-02	2.35E-02
Cholesteryl Esters in HDL	1.01E+00	2.53E-01
Cholesteryl Esters in IDL	6.13E-01	1.59E-01
Cholesteryl Esters in LDL	1.25E+00	3.19E-01
Cholesteryl Esters in Large HDL	2.27E-01	1.30E-01
Cholesteryl Esters in Large LDL	8.20E-01	2.06E-01
Cholesteryl Esters in Large VLDL	5.09E-02	2.31E-02
Cholesteryl Esters in Medium HDL	4.02E-01	9.74E-02
Cholesteryl Esters in Medium LDL	3.01E-01	8.66E-02
Cholesteryl Esters in Medium VLDL	9.10E-02	3.81E-02
Cholesteryl Esters in Small HDL	3.25E-01	4.67E-02

Cholesteryl Esters in Small LDL	1.29E-01	3.28E-02
Cholesteryl Esters in Small VLDL	9.73E-02	3.29E-02
Cholesteryl Esters in VLDL	4.27E-01	1.43E-01
Cholesteryl Esters in Very Large HDL	5.82E-02	2.77E-02
Cholesteryl Esters in Very Large VLDL	2.98E-02	1.46E-02
Cholesteryl Esters in Very Small VLDL	1.27E-01	3.52E-02
VLDL Cholesterol	7.17E-01	2.46E-01
Clinical LDL Cholesterol	2.51E+00	7.27E-01
Cholesterol in Chylomicrons and Extremely Large VLDL	5.67E-02	4.28E-02
Cholesterol in IDL	8.32E-01	2.14E-01
Cholesterol in Large HDL	2.94E-01	1.67E-01
Cholesterol in Large LDL	1.11E+00	2.81E-01
Cholesterol in Large VLDL	9.72E-02	4.71E-02
Cholesterol in Medium HDL	4.88E-01	1.21E-01
Cholesterol in Medium LDL	4.18E-01	1.16E-01
Cholesterol in Medium VLDL	1.69E-01	6.54E-02
Cholesterol in Small HDL	4.39E-01	6.03E-02
Cholesterol in Small LDL	1.78E-01	4.38E-02
Cholesterol in Small VLDL	1.55E-01	5.11E-02
Cholesterol in Very Large HDL	8.18E-02	3.47E-02
Cholesterol in Very Large VLDL	5.36E-02	2.85E-02
Cholesterol in Very Small VLDL	1.85E-01	4.93E-02
LDL Cholesterol	1.71E+00	4.34E-01
HDL Cholesterol	1.30E+00	3.23E-01
Remnant Cholesterol Non HDL Non LDL Cholesterol	1.55E+00	4.15E-01
Total Cholesterol	4.56E+00	9.36E-01
Total Cholesterol Minus HDL C	3.26E+00	8.35E-01
Total Free Cholesterol	1.25E+00	2.68E-01
Total Esterified Cholesterol	3.31E+00	6.73E-01
Total Fatty Acids	1.18E+01	2.39E+00
Saturated Fatty Acids	4.04E+00	9.51E-01
Omega 6 Fatty Acids	4.45E+00	6.81E-01
Linoleic Acid	3.41E+00	6.85E-01
Monounsaturated Fatty Acids	2.82E+00	8.19E-01
Docosahexaenoic Acid	2.34E-01	8.28E-02
Polyunsaturated Fatty Acids	4.98E+00	8.02E-01
Omega 3 Fatty Acids	5.26E-01	2.19E-01
Citrate	6.21E-02	1.22E-02
Lactate	3.81E+00	1.09E+00
Glucose	3.57E+00	1.14E+00
Pyruvate	7.83E-02	3.26E-02
Phosphatidylcholines	2.08E+00	3.74E-01
Phosphoglycerides	2.25E+00	3.97E-01

Phospholipids in Chylomicrons and Extremely Large VLDL	3.63E-02	3.20E-02
Phospholipids in HDL	1.54E+00	3.17E-01
Phospholipids in IDL	2.90E-01	6.59E-02
Phospholipids in LDL	5.98E-01	1.39E-01
Phospholipids in Large HDL	3.21E-01	1.51E-01
Phospholipids in Large LDL	3.53E-01	8.20E-02
Phospholipids in Large VLDL	6.59E-02	3.91E-02
Phospholipids in Medium HDL	4.82E-01	9.96E-02
Phospholipids in Medium LDL	1.59E-01	4.15E-02
Phospholipids in Medium VLDL	1.27E-01	4.73E-02
Phospholipids in Small HDL	6.55E-01	9.23E-02
Phospholipids in Small LDL	8.70E-02	1.84E-02
Phospholipids in Small VLDL	9.61E-02	2.98E-02
Phospholipids in VLDL	4.69E-01	1.80E-01
Phospholipids in Very Large HDL	7.91E-02	4.45E-02
Phospholipids in Very Large VLDL	3.88E-02	2.60E-02
Phospholipids in Very Small VLDL	1.05E-01	2.62E-02
Sphingomyelins	4.42E-01	7.16E-02
Total Cholines	2.53E+00	4.10E-01
Total Phospholipids in Lipoprotein Particles	2.89E+00	4.70E-01
Total Triglycerides	1.30E+00	5.73E-01
Triglycerides in Chylomicrons and Extremely Large VLDL	1.38E-01	1.33E-01
Triglycerides in HDL	1.44E-01	4.72E-02
Triglycerides in IDL	9.90E-02	2.60E-02
Triglycerides in LDL	1.44E-01	4.08E-02
Triglycerides in Large HDL	3.01E-02	1.21E-02
Triglycerides in Large LDL	9.61E-02	2.54E-02
Triglycerides in Large VLDL	1.65E-01	9.14E-02
Triglycerides in Medium HDL	5.38E-02	1.90E-02
Triglycerides in Medium LDL	3.24E-02	1.03E-02
Triglycerides in Medium VLDL	2.70E-01	1.14E-01
Triglycerides in Small HDL	5.25E-02	1.75E-02
Triglycerides in Small LDL	1.53E-02	5.69E-03
Triglycerides in Small VLDL	1.56E-01	6.00E-02
Triglycerides in VLDL	9.09E-01	4.80E-01
Triglycerides in Very Large HDL	7.06E-03	2.69E-03
Triglycerides in Very Large VLDL	1.11E-01	8.16E-02
Triglycerides in Very Small VLDL	6.86E-02	2.12E-02
Creatinine	6.61E-02	1.51E-02
Albumin	3.89E+01	3.36E+00
Glycoprotein Acetyls	7.92E-01	1.17E-01

Units of NMR metabolomics measurement are mmol/l.

Supplementary Table S3: Overall association of 167 metabolic biomarkers with ischemic heart disease

Metabolites	Estimate	p value	p.fdr
Glycoprotein Acetyls	0.213	1.43E-08	1.19E-06
Albumin	-0.195	1.75E-07	9.72E-06
Creatinine	0.087	9.82E-07	4.10E-05
Triglycerides in Very Small VLDL	0.146	5.45E-05	1.01E-03
Triglycerides in Very Large VLDL	0.054	1.42E-01	3.53E-01
Triglycerides in Very Large HDL	0.109	2.30E-03	2.40E-02
Triglycerides in VLDL	0.057	1.27E-01	3.42E-01
Triglycerides in Small VLDL	0.092	1.57E-02	7.85E-02
Triglycerides in Small LDL	0.104	3.06E-03	2.84E-02
Triglycerides in Small HDL	0.080	4.04E-02	1.53E-01
Triglycerides in Medium VLDL	0.042	2.75E-01	4.79E-01
Triglycerides in Medium LDL	0.140	6.51E-05	1.09E-03
Triglycerides in Medium HDL	0.033	3.99E-01	5.62E-01
Triglycerides in Large VLDL	0.036	3.37E-01	5.26E-01
Triglycerides in Large LDL	0.162	5.44E-06	1.82E-04
Triglycerides in Large HDL	0.044	2.71E-01	4.79E-01
Triglycerides in LDL	0.149	2.25E-05	4.69E-04
Triglycerides in IDL	0.159	1.04E-05	2.48E-04
Triglycerides in HDL	0.058	1.29E-01	3.42E-01
Triglycerides in Chylomicrons and Extremely Large VLDL	0.046	1.96E-01	4.11E-01
Total Triglycerides	0.070	5.85E-02	2.08E-01
Total Phospholipids in Lipoprotein Particles	-0.025	5.56E-01	6.83E-01
Total Cholines	-0.045	2.86E-01	4.83E-01
Sphingomyelins	0.007	8.71E-01	9.26E-01
Phospholipids in Very Small VLDL	0.130	1.31E-03	1.68E-02
Phospholipids in Very Large VLDL	0.057	1.38E-01	3.53E-01
Phospholipids in Very Large HDL	0.045	3.47E-01	5.32E-01
Phospholipids in VLDL	0.067	9.46E-02	2.98E-01
Phospholipids in Small VLDL	0.063	1.27E-01	3.42E-01
Phospholipids in Small LDL	0.063	1.22E-01	3.42E-01
Phospholipids in Small HDL	-0.096	1.47E-02	7.73E-02
Phospholipids in Medium VLDL	0.029	4.94E-01	6.30E-01
Phospholipids in Medium LDL	0.006	8.89E-01	9.29E-01
Phospholipids in Medium HDL	-0.100	1.88E-02	8.74E-02
Phospholipids in Large VLDL	0.061	1.24E-01	3.42E-01
Phospholipids in Large LDL	-0.033	4.43E-01	5.97E-01
Phospholipids in Large HDL	-0.045	3.64E-01	5.38E-01
Phospholipids in LDL	-0.008	8.42E-01	9.07E-01
Phospholipids in IDL	0.005	9.07E-01	9.36E-01

Phospholipids in HDL	-0.085	6.00E-02	2.09E-01
Phospholipids in Chylomicrons and Extremely Large VLDL	0.048	1.97E-01	4.11E-01
Phosphoglycerides	-0.035	4.00E-01	5.62E-01
Phosphatidylcholines	-0.057	1.84E-01	4.11E-01
Pyruvate	0.019	5.93E-01	7.07E-01
Glucose	0.185	5.26E-21	8.78E-19
Lactate	0.025	5.11E-01	6.47E-01
Citrate	0.141	8.08E-06	2.25E-04
Omega 3 Fatty Acids	-0.108	1.17E-02	6.83E-02
Polyunsaturated Fatty Acids	-0.067	1.10E-01	3.34E-01
Docosahexaenoic Acid	-0.126	4.11E-03	3.27E-02
Monounsaturated Fatty Acids	0.088	1.33E-02	7.42E-02
Linoleic Acid	-0.039	3.56E-01	5.36E-01
Omega 6 Fatty Acids	-0.044	2.91E-01	4.86E-01
Saturated Fatty Acids	0.057	1.20E-01	3.42E-01
Total Fatty Acids	0.038	3.11E-01	5.09E-01
Total Esterified Cholesterol	-0.050	2.46E-01	4.71E-01
Total Free Cholesterol	-0.019	6.60E-01	7.71E-01
Total Cholesterol Minus HDL C	-0.011	7.93E-01	8.77E-01
Total Cholesterol	-0.042	3.36E-01	5.26E-01
Remnant Cholesterol (Non HDL, Non LDL Cholesterol)	0.003	9.49E-01	9.72E-01
HDL Cholesterol	-0.109	2.37E-02	1.02E-01
LDL Cholesterol	-0.024	5.78E-01	6.99E-01
Cholesterol in Very Small VLDL	0.040	3.56E-01	5.36E-01
Cholesterol in Very Large VLDL	0.051	2.10E-01	4.32E-01
Cholesterol in Very Large HDL	0.038	4.30E-01	5.83E-01
Cholesterol in Small VLDL	0.062	1.41E-01	3.53E-01
Cholesterol in Small LDL	0.002	9.61E-01	9.78E-01
Cholesterol in Small HDL	-0.125	1.57E-03	1.88E-02
Cholesterol in Medium VLDL	-0.007	8.78E-01	9.28E-01
Cholesterol in Medium LDL	-0.017	6.91E-01	7.95E-01
Cholesterol in Medium HDL	-0.130	3.63E-03	3.03E-02
Cholesterol in Large VLDL	0.049	2.29E-01	4.59E-01
Cholesterol in Large LDL	-0.030	4.78E-01	6.17E-01
Cholesterol in Large HDL	-0.049	3.35E-01	5.26E-01
Cholesterol in IDL	-0.051	2.52E-01	4.74E-01
Cholesterol in Chylomicrons and Extremely Large VLDL	0.050	1.90E-01	4.11E-01
Clinical LDL Cholesterol	-0.034	4.23E-01	5.78E-01
VLDL Cholesterol	0.045	2.84E-01	4.83E-01
Cholesteryl Esters in Very Small VLDL	0.014	7.60E-01	8.46E-01
Cholesteryl Esters in Very Large VLDL	0.046	2.72E-01	4.79E-01
Cholesteryl Esters in Very Large HDL	0.024	6.19E-01	7.33E-01
Cholesteryl Esters in VLDL	0.037	3.88E-01	5.54E-01

Cholesteryl Esters in Small VLDL	0.076	7.40E-02	2.42E-01
Cholesteryl Esters in Small LDL	0.009	8.37E-01	9.07E-01
Cholesteryl Esters in Small HDL	-0.147	1.99E-04	3.03E-03
Cholesteryl Esters in Medium VLDL	-0.027	5.34E-01	6.67E-01
Cholesteryl Esters in Medium LDL	-0.009	8.19E-01	8.94E-01
Cholesteryl Esters in Medium HDL	-0.137	2.18E-03	2.40E-02
Cholesteryl Esters in Large VLDL	0.043	3.05E-01	5.04E-01
Cholesteryl Esters in Large LDL	-0.023	5.89E-01	7.07E-01
Cholesteryl Esters in Large HDL	-0.056	2.65E-01	4.79E-01
Cholesteryl Esters in LDL	-0.016	6.99E-01	7.95E-01
Cholesteryl Esters in IDL	-0.059	1.84E-01	4.11E-01
Cholesteryl Esters in HDL	-0.123	1.05E-02	6.83E-02
Cholesteryl Esters in Chylomicrons and Extremely Large VLDL	0.057	1.41E-01	3.53E-01
Concentration of Very Small VLDL Particles	0.100	1.60E-02	7.85E-02
Concentration of Very Large VLDL Particles	0.060	1.19E-01	3.42E-01
Concentration of Very Large HDL Particles	0.056	2.31E-01	4.59E-01
Concentration of VLDL Particles	0.076	6.21E-02	2.12E-01
Concentration of Small VLDL Particles	0.089	2.60E-02	1.09E-01
Concentration of Small LDL Particles	0.033	4.23E-01	5.78E-01
Concentration of Small HDL Particles	-0.119	2.68E-03	2.64E-02
Concentration of Medium VLDL Particles	0.026	5.35E-01	6.67E-01
Concentration of Medium LDL Particles	-0.005	9.08E-01	9.36E-01
Concentration of Medium HDL Particles	-0.121	6.41E-03	4.87E-02
Concentration of Large VLDL Particles	0.056	1.54E-01	3.74E-01
Concentration of Large LDL Particles	0.045	2.86E-01	4.83E-01
Concentration of Large HDL Particles	-0.044	3.86E-01	5.54E-01
Concentration of LDL Particles	0.031	4.61E-01	6.06E-01
Concentration of IDL Particles	-0.011	8.02E-01	8.81E-01
Concentration of HDL Particles	-0.124	3.58E-03	3.03E-02
Concentration of Chylomicrons and Extremely Large VLDL Particles	0.051	1.63E-01	3.83E-01
Free Cholesterol in Very Small VLDL	0.097	2.10E-02	9.43E-02
Free Cholesterol in Very Large VLDL	0.053	1.73E-01	4.01E-01
Free Cholesterol in Very Large HDL	0.080	7.04E-02	2.35E-01
Free Cholesterol in VLDL	0.053	1.95E-01	4.11E-01
Free Cholesterol in Small VLDL	0.038	3.74E-01	5.47E-01
Free Cholesterol in Small LDL	-0.016	7.09E-01	8.00E-01
Free Cholesterol in Small HDL	-0.044	2.70E-01	4.79E-01
Free Cholesterol in Medium VLDL	0.019	6.49E-01	7.63E-01
Free Cholesterol in Medium LDL	-0.035	4.06E-01	5.64E-01
Free Cholesterol in Medium HDL	-0.099	2.78E-02	1.11E-01
Free Cholesterol in Large VLDL	0.053	1.85E-01	4.11E-01
Free Cholesterol in Large LDL	-0.050	2.51E-01	4.74E-01
Free Cholesterol in Large HDL	-0.021	6.69E-01	7.76E-01

Free Cholesterol in LDL	-0.043	3.21E-01	5.15E-01
Free Cholesterol in IDL	-0.026	5.46E-01	6.76E-01
Free Cholesterol in HDL	-0.053	2.67E-01	4.79E-01
Free Cholesterol in Chylomicrons and Extremely Large VLDL	0.042	2.69E-01	4.79E-01
Total Lipids in Very Small VLDL	0.104	1.19E-02	6.83E-02
Total Lipids in Very Large VLDL	0.055	1.47E-01	3.61E-01
Total Lipids in Very Large HDL	0.048	3.16E-01	5.12E-01
Total Lipids in VLDL	0.059	1.29E-01	3.42E-01
Total Lipids in Small VLDL	0.084	3.77E-02	1.46E-01
Total Lipids in Small LDL	0.031	4.47E-01	5.98E-01
Total Lipids in Small HDL	-0.096	1.48E-02	7.73E-02
Total Lipids in Medium VLDL	0.031	4.56E-01	6.04E-01
Total Lipids in Medium LDL	0.001	9.81E-01	9.85E-01
Total Lipids in Medium HDL	-0.110	1.15E-02	6.83E-02
Total Lipids in Lipoprotein Particles	0.001	9.77E-01	9.85E-01
Total Lipids in Large VLDL	0.046	2.44E-01	4.71E-01
Total Lipids in Large LDL	-0.016	7.14E-01	8.00E-01
Total Lipids in Large HDL	-0.044	3.79E-01	5.50E-01
Total Lipids in LDL	-0.006	8.90E-01	9.29E-01
Total Lipids in IDL	-0.017	6.99E-01	7.95E-01
Total Lipids in HDL	-0.090	5.21E-02	1.89E-01
Total Lipids in Chylomicrons and Extremely Large VLDL	0.047	1.91E-01	4.11E-01
Total Concentration of Lipoprotein Particles	-0.114	7.28E-03	5.29E-02
Average Diameter for VLDL Particles	-0.048	1.62E-01	3.83E-01
Average Diameter for LDL Particles	0.066	9.27E-02	2.98E-01
Average Diameter for HDL Particles	0.009	8.62E-01	9.22E-01
Apolipoprotein B	0.030	4.80E-01	6.17E-01
Apolipoprotein A1	-0.107	1.66E-02	7.90E-02
Total Concentration of Branched Chain Amino Acids (Leucine + Isoleucine + Valine)	0.027	4.79E-01	6.17E-01
Tyrosine	0.121	1.03E-03	1.44E-02
Phenylalanine	0.048	2.70E-02	1.10E-01
Valine	0.036	3.60E-01	5.36E-01
Isoleucine	0.044	2.43E-01	4.71E-01
Leucine	0.001	9.85E-01	9.85E-01
Alanine	0.093	9.22E-03	6.41E-02
Glycine	0.029	5.71E-01	6.96E-01
Histidine	-0.052	2.14E-01	4.36E-01
Glutamine	0.036	3.44E-01	5.32E-01
Acetone	0.047	9.97E-02	3.08E-01
Acetoacetate	0.067	2.15E-02	9.43E-02
Acetate	0.040	4.52E-02	1.68E-01
X3 Hydroxybutyrate	0.079	1.11E-02	6.83E-02

Supplementary Table S4: Association of 10 metabolic biomarkers with red meat consumption and with ischemic heart disease in women

(a) Unprocessed red meat consumption

Metabolites	Beta,95% CI_meat	p_meat	OR,95% CI_IHD	p_IHD
Concentration of Very Small VLDL Particles	0.03 (0.02, 0.04)	2.19E-15	0.96 (0.8, 1.13)	6.11E-01
Phospholipids in Very Small VLDL	0.03 (0.02, 0.03)	6.89E-13	0.99 (0.83, 1.17)	9.34E-01
Total Lipids in Very Small VLDL	0.03 (0.02, 0.04)	1.74E-15	0.96 (0.81, 1.14)	6.84E-01
Triglycerides in IDL	0.02 (0.01, 0.03)	8.78E-10	1.04 (0.88, 1.23)	6.10E-01
Triglycerides in Large LDL	0.02 (0.01, 0.03)	1.91E-09	1.03 (0.87, 1.22)	6.90E-01
Triglycerides in LDL	0.02 (0.01, 0.03)	9.16E-09	1.03 (0.86, 1.21)	7.44E-01
Triglycerides in Medium LDL	0.02 (0.01, 0.02)	5.13E-07	1.02 (0.85, 1.21)	7.94E-01
Triglycerides in Small LDL	0.02 (0.01, 0.02)	2.22E-07	1.01 (0.84, 1.19)	9.31E-01
Triglycerides in Very Large HDL	0.01 (0, 0.02)	2.74E-03	0.99 (0.82, 1.16)	8.73E-01
Triglycerides in Very Small VLDL	0.02 (0.01, 0.03)	4.70E-09	1.07 (0.9, 1.25)	4.40E-01

(b) Processed meat consumption

Metabolites	Beta,95% CI_meat	p_meat	OR,95% CI_IHD	p_IHD
Concentration of Very Small VLDL Particles	0.02 (0.01, 0.03)	3.36E-08	0.96 (0.8, 1.13)	6.11E-01
Phospholipids in Very Small VLDL	0.02 (0.01, 0.03)	4.93E-08	0.99 (0.83, 1.17)	9.34E-01
Total Lipids in Very Small VLDL	0.02 (0.01, 0.03)	3.72E-08	0.96 (0.81, 1.14)	6.84E-01
Triglycerides in IDL	0.02 (0.02, 0.03)	2.08E-09	1.04 (0.88, 1.23)	6.10E-01
Triglycerides in Large LDL	0.02 (0.02, 0.03)	2.98E-09	1.03 (0.87, 1.22)	6.90E-01
Triglycerides in LDL	0.03 (0.02, 0.03)	4.90E-11	1.03 (0.86, 1.21)	7.44E-01
Triglycerides in Medium LDL	0.03 (0.02, 0.03)	6.54E-12	1.02 (0.85, 1.21)	7.94E-01
Triglycerides in Small LDL	0.03 (0.02, 0.04)	7.71E-17	1.01 (0.84, 1.19)	9.31E-01
Triglycerides in Very Large HDL	0.02 (0.01, 0.02)	6.49E-05	0.99 (0.82, 1.16)	8.73E-01
Triglycerides in Very Small VLDL	0.03 (0.02, 0.04)	2.21E-12	1.07 (0.9, 1.25)	4.40E-01

Supplementary Figure S1: Flow chart of eligible study participants in analyses

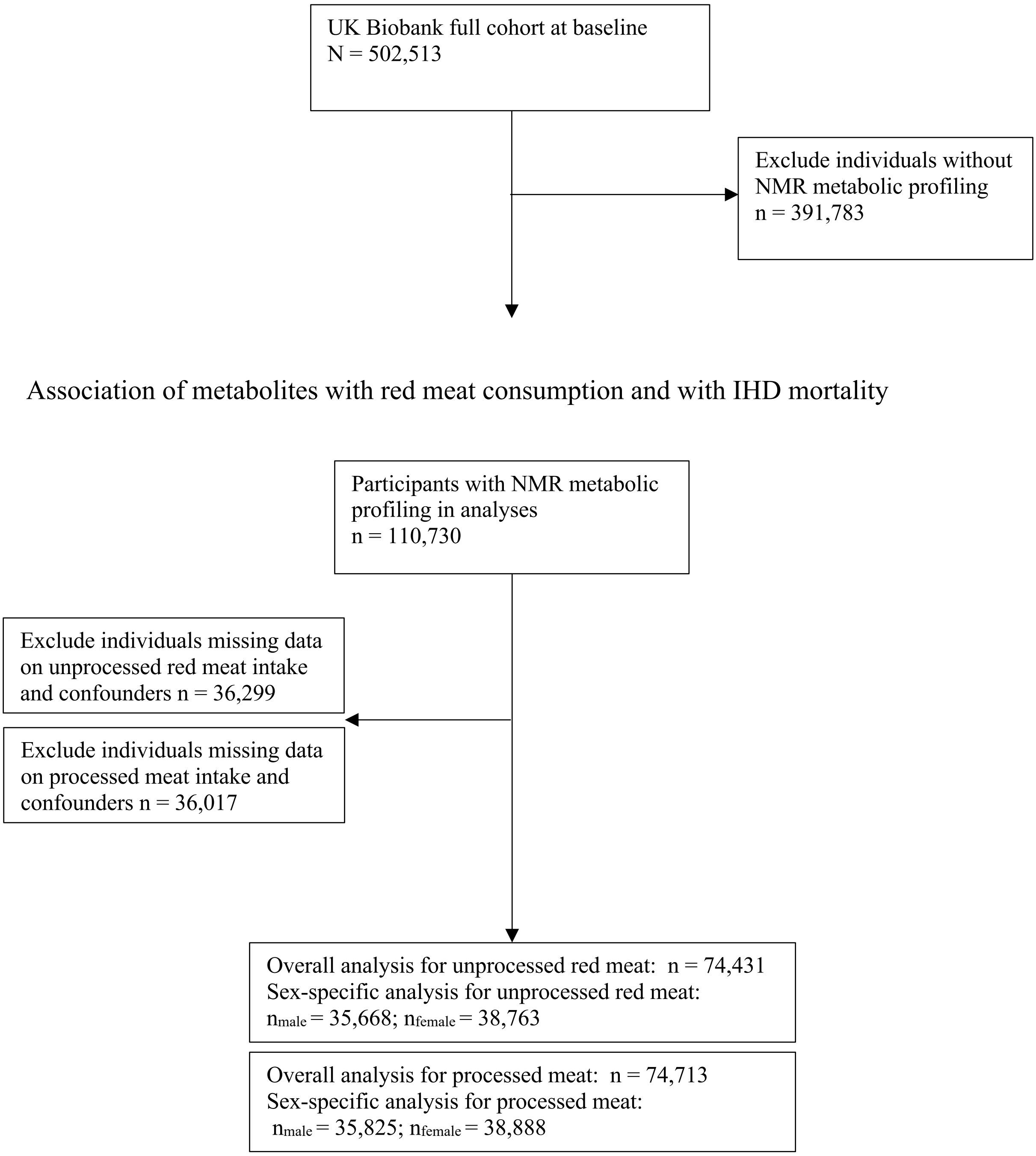

Supplement: Supplementary file 1 [file nutrients-15-01865-s001.zip › Supplementary.pdf]
