# Supplementary figures and images for: Exploration of Metabolic Biomarkers Linking Red Meat Consumption to Ischemic Heart Disease Mortality in the UK Biobank
Source: Nutrients. 2023 Apr 13;15(8):1865. doi: 10.3390/nu15081865 (PMC10142709; doi:10.3390/nu15081865)

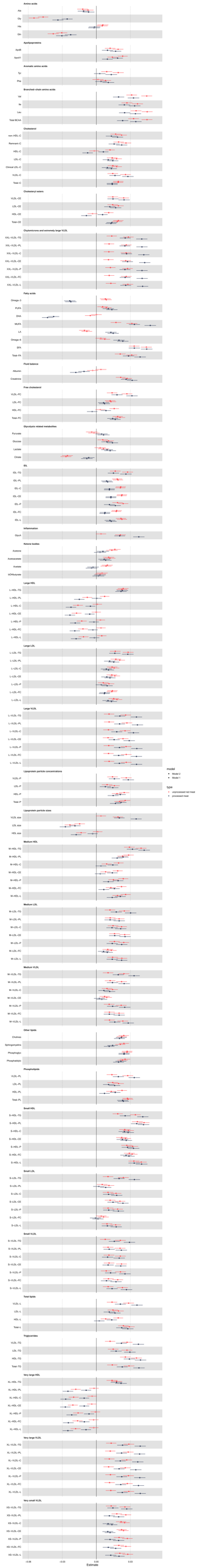

Supplement: Supplementary file 1 [file nutrients-15-01865-s001.zip › Supplementary Figure S2.pdf]
